# The Mediating Effects of Coping Style on the Effects of Breath Count Mindfulness Training on Depressive Symptoms among International Students in China

**DOI:** 10.1155/2020/8859251

**Published:** 2020-08-28

**Authors:** Simeng Gu, Yawen Li, Fei Liang, Rou Feng, Zhi Zeng, Fushun Wang

**Affiliations:** ^1^Institute of Brain and Psychological Sciences, Sichuan Normal University, Chengdu, China 610060; ^2^Department of Medical Psychology, Jiangsu University Medical School, Zhenjiang 210023, China; ^3^School of Health Economics and Management, Nanjing University Of Chinese Medicine, Nanjing, China

## Abstract

Mindfulness training has gained popularity in the scientific field and has been proposed as an efficient way for emotional regulation. Mindfulness-based cognitive therapy (MBCT) is designed especially for depressive people in reducing risk of depression relapse and is recommended in national guidelines as a treatment choice for relapse prevention in recurrent depression. The aim of the current study was to investigate the effects of mindfulness training on depressive symptoms of international students and probe into the mediating role of mindfulness in stressful events and depression. In addition, we introduced a new kind of mindfulness training, the breathing exercise-based mindfulness training, which is based on the integration of Buddhism and Daoism. Self-report questionnaires assessing the coping style, abnormal depressive behavior, and stressful live events were completed in 260 international students in China (mean age = 21.4 years). The results showed that (1) many international students showed depression symptoms, (2) stressful life events play a completely mediating role in the initiation of depression and anxiety, and (3) mindfulness training for 8 weeks significantly reduced the depressive symptoms, and it was also related to a positive coping style. This study has certain theoretical significance in exploring the mechanism of the occurrence and development of depression among international students and provides useful tools for this special group of international students. In addition, the international students can also learn Chinese culture through the training. These findings indicate that mindfulness training and positive coping style are interrelated with treating depressive symptoms for international students.

## 1. Introduction

With the growing influence of Chinese education, the number of students studying in China continues to increase. In 2017, China was already the destination for the largest population of overseas students in Asia [1]. Mental health problems of these international students caused by cultural shocks have gradually become the focus of education management. Studies have shown that there are many emotional problems among these students, such as loneliness, anxiety, and depression [2]. Depression is a worldwide emotional disorder that affects an individual's adjustment to school [3, 4] and sometimes leads to self-harm or suicide. Depression also increases the risk of cardiovascular disease, raising the death rate for depressed people to 80% [5]. Therefore, it is an important task to deal with the mental health of overseas students in China, to understand their depressive mood states, especially to explore the causes of their depression.

Even though depression is the leading cause of disability worldwide [6], there is no efficient treatment for this disease, for the underlying neural mechanism of emotion is unclear [7]. The most widely accepted theory about affective disorders is the monoamine theory, which suggests that monoamines, including norepinephrine, dopamine, and serotonin (5-HT), are the major reasons for emotion disorders [8], and the first line of treatment for depression also targets these monoamines [9]. However, psychological therapy has also been used for depressive disorders, such as mindfulness-based cognitive therapy (MBCT) [10]. Mindfulness is a kind of meditation originating from an Eastern Buddhist document and was introduced to the Western culture by Kabat-Zinn [11]. Later practices have given rise to many different mindfulness training programs, including mindfulness-based cognitive therapy (MBCT) [12], which is specially developed to psychologically treat individuals at risk of depressive relapse [13].

MBCT was described as a “program that focuses on learning how to mindfully attend to body sensations through the use of body scans, gentle stretching, and yoga mindfulness exercises, along with discussion and practices geared toward applying mindfulness” [14, 15]. MBCT trains an individual's awareness and acceptance of both negative and positive emotions [16], being awarene to daily life experiences, including coping with stress [17]. MBCT was designed originally as a relapse prevention for people with a history of recurrent depression ^[^18^,^^19^^]^; it is not clear if MBCT can be used to people with current diagnosis of depressive disorders. Mindfulness invites participants to bring their full awareness to current experiences, but people with a current stressful life are likely to experience negative emotional feelings [20]. In addition, previously, we have shown that a negative coping style is related to stress-induced mood disorders such as anxiety and depression [21]; there are few studies about mindfulness with coping style in people under stress during their depressive episodes.

Even though some studies have suggested to use MBCT to people who are experiencing a current episode of depressive disorders [22], majority of research has focused on clinical intervention studies to evaluate the efficacy of mindfulness therapy on normal populations [23]. Here, we try to use mindfulness training in the international students. International students represent a special group of people, who just moved from their homeland and need to adapt the new environment. And their major problem is getting accustomed to the new environment, just like what mindfulness proposed to bring their full awareness to current experiences. The current study is aimed at exploring the effect of mindfulness training on depression and the mediating role of coping style between stressful event and depression. It also sought to identify the mediating role of coping style between mindfulness training and depression. It was hypothesized that mindfulness training can help the depressive international students adapt to school life, and coping style played a partial mediating role between depression and mindfulness training.

## 2. Methods

### 2.1. Participants

260 foreign students were recruited from Jiangsu University and Nanjing University of Chinese Medicine. The investigators distributed the questionnaires to the students and explained the instructions, which includes the purpose of the test, the principle of voluntariness, and the anonymous way of answering the questions. 24 students' questionnaires had missing answers or wrong answers, and they were removed, with an effective rate of 90.7%. The Lie scale in EPQ-RSC of the Eysenck Personality Questionnaire, was further adopted. The study showed that the reliability and validity of the L subscale were relatively high, which reached the requirements of psychometrics [24]. The total effective students' questionnaires were 213, with an effective rate of 90.7% and a total effective rate of 81.92%. Among them, 112 were boys and 101 were girls.

All the procedures are done according to the ethic assessment sessions taking place in class during school time, supervised by members of the research team. The survey lasted half an hour, and the questionnaires were collected on the spot. The research was approved by the Human Research Ethics Committee of Jiangsu University. Information about the study was provided, and informed consent and assent were obtained from each participant.

### 2.2. Research Tools

#### 2.2.1. Self-Rating Depression Scale (SDS)

The self-rating depression scale (SDS) compiled by Zung (1965) was used to measure the students' depression level [25]. There are 20 items in this scale, and each item is scored on a scale of 4. The higher the score means the higher the degree of depression. This scale is easy to operate, and the score is not affected by age, gender, economic status, and other factors. It is one of the commonly used self-measuring scales for depression. In this study, the confirmatory factor analysis results of the questionnaire showed good structural validity, and the fitting indicators were as follows: *x*^2^ = 1.903, df = 1, TLI = 0.950, GFI = 0.994, CFI = 0.992, and RMSEA = 0.077. In this study, Cronbach's *α* is 0.76.

#### 2.2.2. The Life Event Test

The life event test includes 48 most popular life events, which includes three aspects: family life (28), work and study life (13), and social and friends (7), and 2 empty questions. The score ranges from 1 to 4 depending on its effects on the person's mind and also the effect duration on his mind, for example, from one month to half a year.

#### 2.2.3. The Simple Coping Style Questionnaire

The Simple Coping Style Questionnaire (SCSQ) contains 20 items assessing the coping style, scores 0 (never) to 3 (always). Items 1-12 belong to positive coping and 13-20 belong to negative coping. If the average difference between positive coping and negative coping is greater than 0, it is positive coping and less than 0 is negative coping. The current study total questionnaire internal consistency *α* = 0.76.

### 2.3. Mindfulness Training and Psychological Therapy

The MBCT training includes one hour of cognitive therapy with the knowledge about depression and mindfulness and also some idea about meditation. Then, they received 20 minutes of audio-guided training in “attention to breath,” based on a mindfulness-based stress reduction program. The participants were required to “focus their attention on sensing the air slowly entering and leaving the nose,” while listening to the audio file, in which they were instructed to relax the body and concentrate on their breath sensations. For the mindfulness training, it is hard for the subjects to concentrate or focus their attention on “here and present.” In order to focus their attention on “here and present,” they can do one kind of breathing exercise: count the numbers from one to ten while breathing in, and then, hold the breath for ten seconds, and then, slowly breathe out. Then, after some training, they can go to an advanced level of breathing exercise: count the numbers 1-10 while breathing in, hold the breath for 10 seconds, then count the numbers 1-10 while breathing in again, and hold the breath for 10 more seconds. They can scan their bodies while holding the breath, thinking the air going to any part of the body they were scanning, such as the foot and leg. Each participant practices at least half an hour a day with the audios.

### 2.4. The Analysis of Salivary Cortisol Contents

The detailed procedures for analyzing salivary cortisol contents were described previously [26]. Briefly, at 9:00 am, the subjects were asked to wash their mouths with distilled water and, 5 minutes later, to put some sterilized absorbent cotton under the tongue to take saliva. 1 ml saliva was dissolved in 50-microliter methanol for cortisol analysis that was done on a Qtrap 3200 liquid chromatography-tandem mass spectrometer (ABI, USA). Cortisol was ionized with atmospheric pressure chemical ionization and identified in the positive ion mode using the multiple reaction monitoring mode. The assay method had good linearity in the range of 0.8-250.0 pg/mg, showing the square coefficient of correlation at 0.999. It also had good sensitivity, accuracy, and precision, showing limits of detection and quantitation at 0.3 and 0.8 pg/mg, and intraday and interday coefficients of variation less than 15% and recovery ranging between 85 and 115% (Chen et al., 2019), which fit the requirements of hair cortisol measurement.

### 2.5. Statistical Analysis

All analyses were performed using SPSS 22.0. All statistical tests were two-sided, and the significance level was set at *p* < 0.05. A paired *t*-test or repeated two-way ANOVA was used to compare differences between groups, and repeated one-way ANOVA was used to test the significant difference among the groups. The general linear regression analysis was used to explore the relations between depression and life events. Asymptotic and resampling strategies were used to examine the mediating role of coping style on the association between depression and stressful life events.

## 3. Results

### 3.1. Descriptive Statistics

The demographic characteristics of the international students in categorical variables are shown in Table 1. 97 students have come to China for less than one year, and 116 have come for more than one year.

The degree of depression was measured by the depression index: depression index = score/full score. Depression index < 0.50 means no depression, 0.50 ≤ depression index ≤ 0.59 was mild depression, 0.60 < depression index ≤ 0.69 was moderate depression, and depression index > 0.70 was severe depression. The mean depression index for all participants was 0.46, which suggests a mild depression. Among the students, 82 have no depression, accounting for 38.49%; 72 have mild depression, accounting for 33.80%; 38 students have moderate depression, accounting for 17.84%; and 11 students with severe depression, accounting for 5.16%. The mean depression degree of male students and female students was 49.93 and 42.55, respectively. On the whole, female students encountered fewer depression problems than male students, but the *p* value was 0.26 > 0.05, showing no significant statistical significance.

### 3.2. Correlation Analysis Life Events and Coping Style and Depression

The correlation analysis results of life event and depression are shown in Table 2. The results showed that depression symptoms were significantly correlated with life events and coping style. The results showed that life events had a significant positive effect on depression and coping style has a significant negative predictive effect on depression. The total score of life events can be regarded as the severity of symptoms. In addition, the subjects can be divided into the no symptom group, suspicious group, and symptom groups according to their scores.

Then, we used depression symptoms as the dependent variable and life events and adaption style as independent variables; the relationships between depression and life events and coping style were calculated. The indirect effect of coping style on the relationship between life events and depression was examined using the SPSS 20.0 PROCESS procedure. The results showed that life events can induce depression (*p* < 0.01, Table 3), and positive coping style also affected depression (Table 3, *p* < 0.01). The results showed that life events have a significant positive predictive effect on depression and coping style has a significant negative predictive effect on depression. The total score of life events can be regarded as the severity of symptoms.

### 3.3. Mindfulness Training on Depression

There are about 110 students who got high depression scores (mild depression and moderate with scores from 0.5 to 0.7) among the total 213 (the 11 students with severely high scores > 0.7 were excluded and suggested to see doctors). The 110 students were randomly divided into three groups, one group of 36 students with normal psychological counseling (narrative therapy by a registered psychological counselor, 2 hours a week, free of charge); 37 students were neglected, telling them they are normal but need more sports, such as half-hour running (once a day); and 37 students were trained with mindfulness training (twice a week, plus self-practice once a day). The results showed that after 8 weeks, both the narrative group and mindfulness training group improved with emotional depression, except the running group. The mindfulness group showed the best result (*p* < 0.01, paired *t*-test, *n* = 36‐37); psychological therapy showed similar therapy results (*p* < 0.01, paired *t*-test, *n* = 36‐37) (Table 4). Further analysis with one-way ANOVA found that the running group shows the least treating results.

Similarly, we measured the salivary cortisol levels before and after the mindfulness training. The present study utilized cortisol content in the saliva as the biomarker of total stress reactivity. Cortisol content was determined with high-performance liquid chromatography-tandem mass spectrometry. The results revealed that salivary cortisol was decreased after mindfulness training (Table 5, *p* < 0.01, repeated one-way ANOVA).

### 3.4. Effects of Mindfulness Training Are Related to Coping Style

We then analyzed the effects of mindfulness on depression with respect to the coping style. Among the 37 subjects in the mindfulness group, 15 (40.5%) showed positive coping, with the positive coping score averaging 5.43 ± 2.54 (*n* = 15), and 22 showed negative coping, with the negative coping score averaging −6.83 ± 3.82 (*n* = 22). And the mindfulness training showed better results for those with positive coping (*p* < 0.01, one-way ANOVA, *n* = 15‐22, Table 6). Consistently, in the narrative therapy group, 19 showed positive coping, with the positive coping score 4.25 ± 3.14 (*n* = 19), and 18 showed negative coping, with the negative coping score averaging −3.14 ± 2.91 (*n* = 18‐19, Table 6). These data suggested that the mindfulness training and the coping style are related; mindfulness training affects coping style.

## 4. Discussion

### 4.1. Relationship between Depression, Life Events, and Coping Style

There are many adaption problems for international students [27, 28]. Previous studies have reported the relationship between homesickness and depression among international students [29]. As far as we know, there are few studies about the mediating effects of coping style on the mindfulness of depression. Previous studies found that self-esteem partially mediated the association between mindfulness and social anxiety in inland undergraduates [30]. And our previous study found that coping style mediated depression with eating disorders in undergraduates too [21]. In this study, we are the first to use mindfulness training on international students and found that it is a good way to help them accommodate the new environment abroad. In addition, the results of correlation analysis showed that depression was significantly correlated with both life event and coping style. The regression results showed that life events had a significant positive effect on depression, and coping style had a significant negative predictive effect on depression. These findings are consistent with previous studies showing that individuals with negative coping style are more likely to have mood disorders such as anxiety and depression [31], highlighting the importance of coping styles established early in life.

### 4.2. Mindfulness with Buddhism and Daoism

MBCT is derived from mindfulness, which was introduced to the medical field by Kabat-Zinn at the University of Masachusettes for the first time in 1980 [32]. The fundamental component of mindfulness is attention, and it is critically important to pay attention to what is happening “right now and here.” Or mindfulness is characterized as paying attention to all stimulations from external senses or internal senses and being purely observing without being involved in the experience [33]. Attention is critical for mindfulness, but it is difficult to sustain. Almost 50% of the time we are awake, our mind is wandering. Here, we used a short breathing exercise to count the numbers while breathing to reduce mind wandering [34].

According to Buddhism, every individual has six “roots” that bother him and make his mind wander. The six roots mean the senses from the “eye, ear, nose, tongue, body, and mind.” One good way to remove them is just focusing on one of them, such as a beautiful scenery or a good taste. Focusing on the body sense, such as feeling the breath, has been regarded as a very good way for Buddhism or Daoism. The breath focusing mindfulness is a process of focusing on slow, diaphragmatic breathing and putting oneself in the “moment” [35]. As reported before, breath counting in mindfulness is associated with more meta-awareness, less mind wandering, better mood, and greater nonattachment (i.e., less attention captured by distractors formerly paired with a reward). By breath exercise, counting the numbers while breathing, and holding the breath, the participants can help focus their attention on the present.

For international students to adapt to the Chinese culture, they also need to learn the Chinese culture. Mindfulness is derived from Buddhism; actually, there are many kinds of trainings in the traditional Chinese culture, such as Daoism, meditation, and Chan. The difference lies in the target of the attention: the normal mindfulness usually focuses attention on the body, like scanning the body from foot to head. The breath count mindfulness pays attention to the breath, which is the major topic for Daoism. Daoism is the basis for Chinese philosophy and Chinese medicine, and it has many kinds of psychological training methods, such as meditation, Taichi, or Qigong, or Gongfu. One key point of the Daoism training is “Lian Qi,” or “Qi gong,” which means to do breathing exercise, to hold the air in the body, like the Gongfu Panda. Actually, everyone is using the Qi every day; for example, when an individual wants to move a heavy object, or tries to hit somebody, or even tries to stretch the body, he would have a deep breath and hold the air inside. In ancient China, the Taoists usually did exercises to keep longevity through external Chinese alchemy, or internal alchemy. External alchemy means using the drugs, while the internal alchemy means doing the breath (Qi) exercise. Breath exercise (Qi Gong) can not only help the individual to direct their attention to the body for signals of “now and here” but also increase the pressure in the belly to exercise the internal organs, such as the stomach and intestine, to increase the blood flow in the organs, or reunite the body and mind and thus the “spirit” [36].

### 4.3. Meditating Role of Copying Style on Mindfulness Training

Massachusetts University Medical Center has adopted more than 10,000 patients in the last 30 years, with each completing the 8-week mindfulness-based stress reduction program. It is reported that every patient who participated in the program liked it and has made a changing point in their lives [37]. And it has reported that various medical syndromes had been markedly decreased after the program, such as depression, anxiety, and sleep disorders [38]. So far, mindfulness training has been used in many medical disorders, such as psoriasis and fibromyalgia, in addition to affective disorders [39].

Mindfulness training may help depressed people gain attention control and reduce the activation of associative networks of negative thoughts, allowing depressive thoughts to enter and leave consciousness without spiraling the individual into depressive rumination [40, 41]. Over time, mindfulness practices may thus reduce the links between depressive thoughts in the associative network [42]. However, mindfulness training is not always effective in depression and anxiety disorders [43]. Previous studies have found inconsistent relationships between trait mindfulness and state mindfulness. The reason might be that the subjects need to have some trait mindfulness and state mindfulness to get good levels of mindfulness experience. Consistently, some studies suggested that self-esteem might mediate this process [44].

Given that depressive patients are characterized by emotional and attention biases as well as negative coping style, this study probed into the role of negative coping style on the effects of mindfulness. And the data in this study found that mindfulness was associated with negative adaptive coping strategies in the face of stress. This is consistent with previous reports that mindfulness-related well-being was mediated by both appraising situations as less stressful and using more adaptive strategies in coping with stress [45]. In all, mindfulness training can help international students get better results for helping them face stressful life events and get greater positive emotions and less negative emotions.

Stress is evolutionarily benefit to all lives, but overwhelming stress is a critical causing factor for many neurological disorders [46]; for example, oxidative stresses are important factors in neural degenerative disorders, possibly due to increased levels of reactive oxygen species. Stress includes both physiological stress and psychological stress, and recently, it is found that even psychological stress can induce mental disorders or neurological disorders [47]. Sometimes, stress can induce very long-lasting changes in the neural circuits underlying emotional regulations, which facilitate some neurological diseases [48]. Mindfulness is a very useful tool not only to serve as a clinical treatment for patients but also to culture a kind of healthy lifestyle. Mindfulness training can also reduce the stress hormone glucocorticosteroids and serotonin and result in effects on stress and depression. In recent years, an increasing interest in mindfulness-based approaches both in clinical application and in the field of research has proved that MBSR is an established program shown to reduce symptoms of stress, anxiety, and depression. Mindfulness is believed to alter emotional response by modifying cognitive-affective processes [49].

Mindfulness has been proved to be an effective way for emotional regulation, which depends on attention transfer and reappraisal. First, mindfulness training helps individuals to pay attention to what is happening right now and here. This is a good way for attention transfer from worries, which are usually memories of the past or worries for the future. Second, mindfulness can also help cognitive reappraisal, because an individual can treat the worries easily when they are relaxed with deep breathing. Previously, we have hypothesized that the human being has three primary kinds of core affects: reward, punishment, and stress [50], and they exclude from each other [50]. The individuals can have a good mood by focusing on positive emotions (reward) to transfer attention from stressful thoughts. MBCT (mindfulness-based cognitive therapy) was designed to help depressive patients, with cognition. Cognition is associated with depression symptoms. Individuals in a major depressive episode often display impairment in cognitive control [51]. Cognitive models of depression posit that negatively biased self-referent processing and attention have important roles in the disorder [52]. Yoon et al. [53] suggested that patients with depression having mild cognitive impairment (MCI) showed poorer cognitive function than nondepressed patients with MCI in some cognitive domains. Improvement in depression was related to improvement or prevention of the decline in cognitive measures. For instance, depressed patients may have persistent negative thoughts; rumination interacts with negative cognitive styles to predict the duration of depressive symptoms; rumination enhances the effects of depressed mood on thinking, making it more likely that people will use the negative thoughts and memories activated by their depressed mood to understand their current circumstances. Depressed people, particularly depressed ruminators, find it difficult to inhibit negative thoughts and tend to choose the wrong cognitive activities to distract themselves [54]. A dysphoric mood is maintained through attention and memory functions biased toward negative information, and these cognitive biases also expose individuals to recurrent depression; Xu et al. [55] indicate that there is a negative bias in automatic visual deviance detection. Ruohonen et al. [56] investigated age- and depression-related alterations in the ERPs to sound intensity changes; depression-related effects were found for sensory gating when controlling for medication status or when only nonmedicated depressed participants were included in the analysis. In the depression group, general overexcitability in the processing of sounds was only observed in nonmedicated participants, so the results highlight the importance of studying nonmedicated groups when searching for biomarkers for depression. In other words, cognitive involvement may influence depression-related effects.

### 4.4. Limitations and Prospects of the Study

There are some shortcomings in this study, which need to be improved in future studies. Mindfulness-based cognitive therapy has been proved to be effective for emotional disorders, such as depression and anxiety disorders. Many reports have found that mindfulness training can help appease emotional arousal. Mindfulness training is sure to affect the nervous system [57]. Mindfulness can affect attention, emotion, and self-consciousness; what is the neural mechanism? What is the neural network that is involved? Neurobiological effects of meditation and mindfulness can be detected in the brain, particularly in areas related to attention and memory, in perception and sensory processing, or in self- and autoregulation, including control of stress and emotions [58]. In addition, monoamine has been proved to be the substrate for emotions [59]; what are the effects of mindfulness training on the monoamines?

## Figures and Tables

**Table 1:** Demographic characteristics and depressive behaviors (*N* = 213).

Variables	*N*	%	Depression (mean ± SD)	*p*
Majors				
Medicine	89	41.78	47.21 ± 15.28	<0.05^∗^
Science & engineering	52	24.41	48.29 ± 16.44	
Art	29	13.61	44.07 ± 14.80	
Liberal arts	43	20.19	42.66 ± 13.22	
Sex				
Female	101	47.42	48.10 ± 17.94	0.265
Male	112	52.58	43.81 ± 14.60	
Hometowns				
Asian area	124	58.21	49.93 ± 11.58	<0.05^∗^
African area	89	41.78	42.55 ± 12.33	

Note: ^∗^*p* < 0.05.

**Table 2:** Related studies on depression and life events.

	*M*	SD	Depression	Life event	Positive coping
Depression	46.29	8.66	1		
Life event	30.54	5.99	0.409^∗^	1	
Positive coping	15.76	4.97	-0.332^∗^	-0.369^∗^	1

Note: the data were analyzed with SPSS, *M* represents mean, and SD is standard deviation. The right three panels are the Peterson correlation. ^∗^*p* < 0.05.

**Table 3:** Linear regression of depression and life event and coping style.

Dependent variable	Independent variables	Regression coefficient	Standard regression coefficient	*R* ^2^	Adjust *R*^2^	*F*	*t*	*p*
Depression	Life events	0.694	0.409	0.167	0.162	30.353	5.509	0.001
Depression	Positive coping	0.583	0.332	0.110	0.104	18.702	4.325	0.002

Note: ^∗∗^*p* < 0.01.

**Table 4:** Effects of mindfulness training on depression.

	Before (mean)	SD	After (mean)	SD	*p*
Narrative	61.71	5.64	43.37	4.87	<0.01^∗^
Mindfulness	63.33	5.27	38.54	4.24	<0.01^∗^
Running	62.26	4.64	56.98	4.79	n.s.^#^

Note: n.s.: not significant, ^∗^*p* < 0.01 with paired *t*-test, ^#^*p* < 0.01, repeated two-way ANOVAs (factor 1: intervention type; factor 2: measurement time).

**Table 5:** Effects of mindfulness training on blood cortisol (nmol/l).

	Before (mean)	SD	After (mean)	SD	*p*
Narrative	9.31	0.35	8.32	0.45	<0.01^∗^
Mindfulness	9.35	0.59	8.48	0.44	<0.01^∗^
Running	9.29	0.48	9.18	0.39	n.s.^#^

Note: n.s.: not significant, ^∗^*p* < 0.01 with paired *t*-test, ^#^*p* < 0.01, repeated one-way ANOVA.

**Table 6:** Effects of mindfulness training affected by the coping style.

Coping style	Before (mindfulness)	After therapy	Before (narrative)	After therapy
Negative coping	64.32 ± 5.18	46.95 ± 6.73	61.28 ± 5.38	44.96 ± 4.68
Positive coping	62.68 ± 4.95	32.91 ± 4.15^∗∗^	62.37 ± 4.79	42.43 ± 5.74

Note: ^∗∗^*p* < 0.01, repeat one-way ANOVA.

## Data Availability

Original data are available if required.
